# Iodine intake in the Swiss population 100 years after the introduction of iodised salt: a cross-sectional national study in children and pregnant women

**DOI:** 10.1007/s00394-023-03287-6

**Published:** 2023-12-23

**Authors:** Lena Fischer, Maria Andersson, Christian Braegger, Isabelle Herter-Aeberli, Swiss Iodine Study Collaborators

**Affiliations:** 1 Nutrition Research Unit, Children’s Research Centre, University Children’s Hospital Zurich - Eleonore Foundation, Steinwiesstrasse 75, 8032 Zurich, Switzerland; 2 Laboratory of Nutrition and Metabolic Epigenetics, Institute of Food, Nutrition and Health, ETH Zurich, Zurich, Switzerland

**Keywords:** Iodine, Iodine deficiency, Urinary iodine concentration, Salt iodisation, Pregnant women, School-age children, Switzerland

## Abstract

**Purpose:**

The Swiss voluntary salt iodisation programme has successfully prevented iodine deficiency for 100 years, but dietary habits are changing and today only one-third of processed foods contain iodised salt. We aimed to monitor the current iodine status in children and pregnant women.

**Methods:**

We conducted a nationwide cross-sectional study in children (6–12 years) and pregnant women and measured the urinary iodine concentration (UIC) in spot urine samples. We estimated the iodine intake using UIC and urinary creatinine concentration (UCC) and determined the prevalence of intakes below the average requirement (AR) using the SPADE method. We measured dried blood spot (DBS) thyroglobulin (Tg), TSH and total T4 in pregnant women.

**Results:**

The median UIC was 127 μg/L (bootstrapped 95% CI 119, 140, *n* = 362) in children and 97 μg/L (bootstrapped 95% CI 90, 106, *n* = 473) in pregnant women. The estimated prevalence of inadequate iodine intake (< 65 μg/day) was 5.4% (bootstrapped 95% CI 0.0, 14.6) in children. Half (47%) of the women consumed iodine-containing multivitamin and mineral supplements (≥ 150 μg/day). Compared to non-users, users had higher median UIC (129 vs. 81 μg/L, *P* < 0.001), lower prevalence of inadequacy (< 160 μg/day; 0.2 vs. 31%) and lower DBS-Tg (23 vs. 29 μg/L, *P* < 0.001). All women were euthyroid.

**Conclusions:**

The Swiss diet and current salt fortification provides adequate iodine intake in children, but not in all pregnant women. Iodine supplements cover the dietary gap in pregnancy but are not universally consumed. Therefore, improved use of iodised salt in processed foods is desired to ensure adequate iodine intake in all population groups.

This trial was registered at clinicaltrials.gov as NCT04524013.

**Supplementary Information:**

The online version contains supplementary material available at 10.1007/s00394-023-03287-6.

## Introduction

Salt iodisation as means to correct and prevent iodine deficiency has been a ground-breaking global public health achievement since it was first introduced in Switzerland in 1922 [1, 2]. Today, iodised salt is used in the majority of Swiss households (> 80%) [3], but a recent market survey shows that only one-third of processed foods (with salt) are produced using iodised salt [4]. The low coverage of iodised salt in industrially produced food products may negatively influence the iodine intake [3].

The iodine status in the Swiss population has been monitored in nationally representative studies every 5 years since 1998–1999 [3, 5–7]. Over the past decade, the iodine intake was adequate and stable in 6–12-year-old children [3, 5–7], although at the lower end of the adequate range [8]. However, studies in adults conducted in 2010–2012 and 2009–2013 reported borderline inadequate iodine intake, particularly in women [9, 10]. Low intake has also been observed in pregnant women [3], lactating women [5] and infants [5, 11]. The situation is similar to several other European countries with voluntary salt iodisation [2, 12].

To improve the overall iodine intake in the Swiss population, the iodine content in salt was increased from 20 to 25 mg/kg in January 2014 (permitted range 20–40 mg/kg [13]). This modestly increased UIC in children but had no effect on the iodine intake in pregnant women [3], possibly due to partial use of iodised salt in processsed foods. Further, studies in Swiss adults suggest that iodised salt contributes only half (54%) of the total iodine intake [14] and that the remaining iodine intake comes from other dietary sources, mainly milk and dairy products [15]. However, dietary habits are changing, and cow’s milk consumption is decreasing [16–18]. A growing proportion of the population is following a vegetarian or vegan diet [19–22] and previous studies suggest that individuals following a vegan diet may be at risk for iodine deficiency [23]. Plant-based milk alternatives are low in iodine unless they are fortified [24] and only 8% of vegan processed foods are produced with iodised salt [4].

We conducted a cross-sectional national study in Switzerland from September 2020 to July 2022 with the objective to assess the present iodine status in school-age children and pregnant women and monitor the current effectiveness of the Swiss salt iodisation programme. We compared the current median urinary iodine concentration (UIC) with national data from 1999, 2004, 2009 and 2015 and evaluated longitudinal trends. We also assessed the impact of dietary iodine supplements on the iodine intake and thyroid function in pregnant women.

## Methods

### Study design

We conducted a cross-sectional study from September 2020 to July 2022 and aimed to obtain a representative national sample of primary school children (6–12 years) and pregnant women (18–44 years) in Switzerland. The study design was consistent with previous national iodine status studies [3, 5–7].

Following the WHO recommendations for iodine surveys [8], we used a two-stage probability proportionate-to-size cluster sampling based on current census data from the Swiss Federal Statistical Office [25]. In this census data, Switzerland is divided into five geographical regions and each of the regions is further subdivided into three strata listing communities with different population sizes (< 10,000, 10,000–99,999 and > 99,999 inhabitants). The number of clusters within each region and stratum was calculated proportional to the population size. Children were initially recruited through schools, but the response rate was low, partly due to restrictions during the COVID19 pandemic. The sampling was therefore extended to also involve paediatric practices. Paediatricians were selected based on the same proportionate-to-size criteria as for the schools. Women were recruited through gynaecologists and obstetric clinics. We aimed for 20 clusters including 35 children per cluster and 20 clusters each including 25 pregnant women. Smaller villages were underrepresented due to the absence of a paediatric or gynaecological practice on the countryside.

### Subjects

We contacted 414 randomly selected primary schools by email. Out of the 183 schools that replied, 7 agreed and 6 schools finally participated. In each school, 2–3 classes were randomly selected, and all children in these classes were invited to participate. Children consenting were enrolled in this study. We enrolled 10–20 children in 4 schools and < 10 children in 2 schools.

We reached paediatricians through posters in the newsletter of the Swiss Paediatric Association (“Pädiatrie Schweiz”) (*n* = 6), through cantonal paediatric associations (*n* = 7) and by active communication with randomly selected paediatricians in clusters with lower participation rate (*n* = 2). Of the 15 paediatricians, 6 enrolled 29–35 children and 9 included < 20 children.

We contacted 214 obstetric/prenatal care clinics/hospitals throughout Switzerland and 22 clinics/hospitals agreed to participate. Of the 22 gynaecologists, 17 enrolled between 20 and 26 pregnant women and 4 agreed to enrol an additional 15 pregnant women, while 5 included < 20 pregnant women.

The inclusion criteria were as follows: (1) residence in Switzerland for ≥ 12 months; (2) general good health at the time of enrolment. Additionally, school-age children should be aged between 6 and < 13 years and pregnant women aged between 18 and 44 years with a healthy, singleton pregnancy. We excluded children and women who self-reported exposure to X-ray/CT/MRI iodine-containing contrast agent or use of iodine-containing medication within the last 6 months.

The sample size of the study was determined to assess the median UIC with 5% precision (95% CI) [26]. Previous studies in Swiss women suggest a sample size of 473 participants are needed to estimate median UIC with 5% precision [27] and at least 400 women are needed to estimate the prevalence of inadequate iodine intake [28]. We aimed to enrol 700 school-age children and 500 pregnant women.

This study was approved by the Cantonal Ethical Committee of the Canton of Zurich, representing all Swiss Cantonal Committees (BASEC2020-00192). For each respective participating school, additional approval was obtained from the respective Cantonal Education and Health Department. Written informed consent was obtained from the pregnant women and from the parents of the school-age children. An oral consent was also obtained from the children. All data were collected coded and registered by subject number, location, age and sex. This study was registered in the Swiss National Clinical Trials Portal as well as at clinicaltrials.gov (NCT04524013). The STROBE guidelines were used for reporting of the study data [29].

### Study procedures

The sample collection period was November 2020 to July 2022 for children and September 2020 to March 2022 for pregnant women. Children were recruited with the help of the school teachers and paediatricians, who were informed about the study procedures and in turn instructed the participants. The teachers and paediatricians distributed a written participant information sheet aimed to the parents explaining the aims and procedure of the study, plus a shorter version of the study information aimed for the children. Children who returned a written informed consent form signed by the parents and orally consented were eligible to participate in this study.

Pregnant women were recruited by the supervising physician in the participating obstetric/prenatal care clinics. Gynaecologists were informed in detail about the study procedures. They distributed the written participant information to eligible participants who were given sufficient time to read and take a decision. Written informed consent was obtained before sampling. Participants received no compensation for their participation.

A questionnaire was given to all participants. It was specifically adapted for each population group and was used to assess the inclusion and exclusion criteria, the use of iodised salt in the household, the consumption of foods rich in native iodine and the consumption of iodine-containing food supplements. Pregnant women were further asked about education, cigarette smoking, number of births, number of children living with the women at home and pregnancy trimester.

Height and weight were measured in all participants using standard anthropometric techniques [30]. For the measurements, subjects took off their shoes, emptied their pockets and wore light indoor clothing. Body height was measured to the nearest 0.1 cm using a portable stadiometer in the children visited in the schools and a wall-mounted stadiometer for all participants at the practices/clinics. Body weight was measured to the nearest 0.1 kg using a digital scale.

A spot urine sample was obtained from all study participants for measurement of UIC and urinary creatinine concentration (UCC). In children, we also measured urinary sodium concentration (UNaC). A repeat spot urine sample was collected in a random subsample of 30% of the subjects [28]. Participants were asked to collect a second spot urine sample within a week of the first urine sample. Urine samples were collected at any time of the day, except from the first morning void. The participants were given a plastic cup and were asked to provide ~ 20 mL of fresh midstream urine. All urine samples were aliquoted (2.0 mL) on the day of sampling and stored at 4 °C in the practices of paediatricians or gynaecologists or the participants home for repeat spot urine samples and sent weekly to the Laboratory of Human Nutrition at ETH Zurich, Switzerland, where they were frozen at − 20 °C until analysis.

We collected a dried blood spot (DBS) sample in pregnant women for determination of thyroglobulin (Tg), thyroid stimulating hormone (TSH) and total thyroxine (TT4). Blood drops (50 μL) were collected by a finger prick directly onto filter paper cards (IDBS-226, Perkin Elmer, CT, USA). The DBS cards were dried at room temperature, placed in sealed plastic bags and stored at 4 °C at the gynaecologist practices and sent weekly to the Laboratory of Human Nutrition at ETH Zurich, Switzerland, where they were frozen at − 20 °C until analysis.

All children provided a household salt sample for measurement of the salt iodine concentration. The subjects were asked to bring a 60 g salt sample (three tablespoons) from their homes in clean plastic bags supplied by the study team. The salt samples were stored frozen at − 20 °C until analysis.

### Biochemical analysis

#### Urinary iodine concentration

We analysed UIC in duplicates using the Pino-modification of the Sandell-Kolthoff method at ETH Zürich, Switzerland [31]. External quality control was ensured by measuring two in-house control urine samples that were added to each analysed plate (Supplementary Table S1). We assessed iodine deficiency and iodine excess by comparing the obtained median UIC to thresholds recommended by WHO (< 100 µg/L and ≥ 300 µg/L for school-age children and < 150 µg/L and ≥ 500 µg/L for pregnant women [8]).

#### Urinary creatinine concentration

UCC was measured in duplicate using the modified Jaffé method [32]. External quality control was ensured by measuring two in-house control urine samples that were added to each plate analysed (Supplementary Table S1).

#### Urinary sodium concentration

UNaC was determined in the spot urine samples using flame atomic absorption spectrometry (AA240FS; Varian Inc., Techtron, Agilent Technologies USA). The results were verified by measuring certified reference material, i.e. Seronorm Trace Elements Urine Levels (Sero, Norway) (Supplementary Table S1).

#### Salt iodine concentration

The iodine concentration in salt was analysed using the Pino-modification of the Sandell–Kolthoff method [31]. Before the analysis, the salt samples were diluted in two steps: 1) 5 g of salt was weighed and nanopure water added up to a total weight of 50 g and the solution was heated for 40 min to dissolve the salt in water; 2) 4 g of the first solution was diluted with nanopure water up to a total weight of 50 g. The second dilution was used to perform the analysis. Two in-house control urine samples were used as external quality control (Supplementary Table S1). Salt iodine concentration was defined as “no iodine” at < 5 mg per kg salt, adequate at 15–40 mg/kg and high at > 40 mg/kg [8].

#### Thyroid function parameters

Tg was measured on DBS with a DBS-Tg enzyme-linked immunosorbent assay (ELISA) [33]. Serum control samples (Liquichek Tumor Marker Control; Bio-Rad, Hercules, CA, USA) were used as standards for the DBS-Tg assays. We used two in-house DBS samples for quality control (Supplementary Table S1). Assay-specific reference ranges for DBS-Tg were used for pregnant women (0.3–43.5 μg/L [34]).

We analysed DBS-TSH and DBS-TT4 in the Swiss Newborn Screening Laboratory at University Children's Hospital Zurich. TSH and TT4 were measured with the use of an automated time-resolved fluoroimmunoassay method (NS2400; Labsystems Diagnostics Oy, Vantaa, Finland) and related Neonatal TSH/T4 kit (Labsystems Diagnostics Oy, Vantaa, Finland). Kit- and lab-specific DBS controls were used for the analysis. To calculate the prevalence of thyroid dysfunction, we used kit-specific reference ranges. For pregnant women in the second and third trimesters, we used the normal reference values defined for DBS-TSH in non-pregnant adults (0.1–3.7 mIU/L). In the first trimester, we lowered the upper limit of the DBS-TSH reference range by 18% to 3.0 mIU/L, as recommended by the American Thyroid Association [35]. For DBS-TT4, we applied the assay-specific reference range for non-pregnant adults (20–130 nmol/L) to women in pregnancy weeks 1–6. We then increased the upper reference limit by 5% per week, starting at week 7 (week 7, 20–136.5 nmol/L; week 8, 20–143 nmol/L; week 9, 20–149.5 nmol/L; week 10, 20–156 nmol/L; week 11, 20–162.5 nmol/L; week 12, 20–169 nmol/L; week 13, 20–175.5 nmol/L; week 14, 20–182 nmol/L; week 15, 20–188.5 nmol/L) [35]. From week 16 until birth, we multiplied the non-pregnant adult reference range by 1.5 and used the resulting range of 30–195 nmol/L [35].

### Statistical analysis

We used Excel 2022 version 16.66.1 (Microsoft, Redmond, WA, USA), SPSS version 28.0.1.1 (IBM, Armonk, NY, USA) and R version R 4.2.1 [36] for data processing and analysis. The primary outcome of this study was spot UIC in children and pregnant women. Secondary outcomes were spot UCC and UNaC in school-age children and thyroid function parameters (DBS-Tg, DBS-TSH and DBS-TT4) in pregnant women.

For pregnant women we excluded UIC data from three clinics (*n* = 55) due to suspected iodine contamination from urine glucose test strips [37]. The median UIC for each of the three clinics was significantly higher than the overall median UIC (*P* < 0.001). Out of all spot urines in pregnant women (sample 1), 5% (*n* = 25) of the UIC values deviated > 3 SD from the overall median UIC (sample 1): 60% of the samples *(n* = 15) were collected in the three clinics. For subjects who collected a second urine sample at home, we replaced the excluded first urine sample with the second sample (*n* = 15) and effectively 40 women were excluded from the data analysis. As previously described [5, 37], UIC data from pregnant women obtained in 2004 may also have been exposed to contamination and we excluded data from this year for the UIC trend analysis.

For each subject, we estimated the daily urinary iodine excretion (UIE) by calculating the UIC:UCC ratio and multiplying the ratio by population-specific reference ranges for daily urinary creatinine excretion (UCE) (Eq. 1a) [38]. For children, we used sex- and body height-specific UCE reference values derived in German children [39]. For pregnant women, we used age- and sex-specific creatinine values based on non-pregnant Swiss women (9.821 mmol/24 h = 1.11 g/24 h) [40]. The daily iodine intake was estimated using Eq. (1b) (assuming 92% of consumed iodine is excreted in the urine [41, 42]). Estimated daily sodium excretion was calculated in the same way as for iodine (Eq. 1a).

1a $${\text{Estimated \,\,UIE }}\left( {\upmu {\text{g}}/{\text{day}}} \right) \, = {\text{ UIC }}\left( {\upmu {\text{g}}/{\text{L}}} \right) \, /{\text{ UCC }}\left( {{\text{g}}/{\text{L}}} \right) \, \times {\text{ UCE \,\, reference \,\, values }}\left( {\upmu {\text{g}}/{\text{day}}} \right),$$

1b $${\text{Iodine \,\,intake }}\left( {\upmu {\text{g}}/{\text{day}}} \right) \, = {\text{ Estimated \,\, UIE }}\left( {\upmu {\text{g}}/{\text{day}}} \right) \, / \, 0.{92}.$$

Iodine intakes > 4000 μg/day (*n* = 5) were excluded from the analysis for pregnant women and sodium excretions > 15,000 mg/day (*n* = 7) were excluded from the analysis for children.

We ran descriptive statistical analysis for all variables. Normality was assessed by visual inspection of histograms and QQ-plots and by testing the distributions of continuous variables against a normal distribution using the Kolmogorov-Smirnov test. Normally distributed continuous data are presented as mean ± SD (95% CI). Nonparametric data are presented as median (IQR) and the 95% CI around the median was obtained using the bootstrap technique (*n* = 1000). Skewed data were transformed [log (*x*)] for data analysis. No outliers were removed from the descriptive data.

Differences were assessed using Wilcoxson signed rank test for paired data (e.g. between the first and second urine samples) and using the Mann-Whitney *U* test for two categories. Group differences for more than two categories were tested using the Kruskal-Wallis ANOVA test followed by Mann-Whitney post hoc tests and Bonferroni correction of the significance level (e.g. longitudinal comparison of UIC in school-age children and pregnant women).

Associations between two nonparametric variables were assessed using the Spearman Rho (*r*_s_) correlation. To assess determinants of UIC, UCC and Tg, we performed multiple linear regression using log data. We included determinants that were significantly associated with UIC, UCC and Tg in Spearman correlation analysis or group testing. Chi-square tests were used to evaluate differences for categorical data. Statistical significance was set at *P* < 0.05.

We estimated the adjusted UIC, UCC, UNaC, the habitual daily iodine intake and urinary sodium excretion as well as the prevalence of inadequate iodine intake using the SPADE method and the package “SPADE-RIVM” (version 4.1.17) [43]. SPADE consists of several steps to obtain the habitual distribution from repeated short-term measurements: (1) observed data are transformed through Box-Cox transformation to an approximately normal distribution; (2) within-person variability is removed, resulting in a shrunken distribution at the transformed scale, and (3) data are transformed back to the original scale using a complex back-transformation [43]. The estimated adjusted UIC, UCC, UNaC, habitual iodine intake and habitual urinary sodium concentrations are presented as the 50th percentile (bootstrapped 95% CI, *n* = 750).

We calculated the prevalence of inadequate and excessive iodine intake using the estimated average requirement (AR)/upper tolerable intake level (UL) cut-point method from the SPADE method [43]. The prevalence was determined on the original scale and the 95% CIs were obtained using the bootstrap technique (*n* = 750). We used the AR from the US National Academy of Medicine (NAM) and the UL from the European Food Safety Authority (EFSA), as previously proposed [44]. NAM provides ARs for children in two age groups, i.e. for 4–8-year-old (65 μg/day) and 9–13-year-old (73 μg/day) [42]. For consistency, we used the AR of 4–8-year-old children (65 μg/day) for all children. The overall interpretation of the results did not change compared to when age-specific AR values were used (data not shown). We applied the UL from EFSA of 11–14-year-old children (450 μg/day) for all children [45]. The EFSA UL for 7–10-year-old children (300 μg/day) is inconsistent with the WHO median UIC threshold indicating excess iodine intake in children (median UIC ≥ 300 μg/L) [8]. For pregnant women we applied the AR by NAM (160 μg/day) [42] and the UL recommended by EFSA (600 μg/day) [45]. Iodine deficiency and excess may be considered a relevant public health concern if the prevalence of iodine intakes below the AR or above the UL exceeds 10% in the study population [8, 46, 47].

## Results

### School‐age children

We enrolled 391 children from 6 schools and 15 paediatricians and examined their eligibility. We excluded 29 subjects as they did not fulfil the inclusion criteria (*n* = 14) or provided no first urine sample (*n* = 15). In total, 362 school-age children were included in the data analysis. Subject characteristics of the children are shown in Table 1. In all clusters, the study sample represents half of the intended sample size, except for the Northwest and Midland region where we enrolled 12% of the intended number of participants. For communities with a population < 10,000 inhabitants only 1/3 of the intended number of subjects were obtained.

**Table 1:** Subject characteristics of school-age children (*n* = 362) and pregnant women (*n* = 513) in the 2020–2022 Swiss national study

	School-age children		Pregnant women	
	Value	*n*	Value	*n*
Male/female (%)	49/51	166/172	–	513
Age (years)^a^	9 (7–10)	361	32 (29–34)	502
Height (cm)	136 (128–145)	356	166 (163–170)	487
Weight (kg)	31 (25–39)	356	63 (57–71)^b^	488
BMI (kg/m^2^)	–	–	22.8 (20.8–25.8)^2^	479
Pregnancy week	–	–	26 (19–32)	489
Trimester 1/2/3/NA (%)	–	–	11/48/36/5	59/246/184/24
Presence of thyroid disorders (%)^c^	0.3	1	7	35
Hypothyroidism	0.3	1	5	28
Hyperthyroidism	0	0	1	4
Other/NA	0	0	1	3
Current thyroxine treatment (%)				
Yes/no/NA	0.3/0/0	1/0/0	5/1/1	27/4/4
Current dietary supplement consumption (%)^c^	16	50	96	471
Containing iodine (%)^d^	0	0	47	221
Current use of iodised salt in household (%)^c^	87	266	86	361
Nutrition pattern (%)		307		439
Omnivorous	99	303	97	424
Vegetarian	1	4	3	15
Vegan	0	0	0	0
Daily consumption (%)^c^		297		343
Bread	66	195	54	184
Dairy products	65	194	67	231
Milk	53	157	56	189
Yoghurt	20	59	24	83
Cheese	16	47	21	70
Abstaining from eating certain foods (%)^c^	16	50	24	101
Meat	2	7	6	27
Fish	5	14	11	46
Eggs	2	7	2	7
Dairy products	4	15	3	15
Bread	0	1	1	3
Other^e^	5	14	6	27

*NA* not available

^a^Values are median (interquartile range), all such values

^b^Before pregnancy, kg

^c^Self-reported

^d^Containing 150–220 μg iodine/day

^e^Including gluten, lactose, sugar, raw meat, pig meat, poultry, sea food, crustaceans, nuts, diverse vegetables

The age of the participating children ranged from 6 to 12 years and the median age was 9 years (IQR 7–10) (Table 1). Eighty-seven per cent of the school-age children reported using iodised salt in their homes. A total of 16% (*n* = 50) reported taking a daily dose of dietary vitamin or mineral supplements, but none took iodine-containing dietary supplements.

#### Urinary concentrations of iodine, sodium and creatinine

**Fig. 1:**
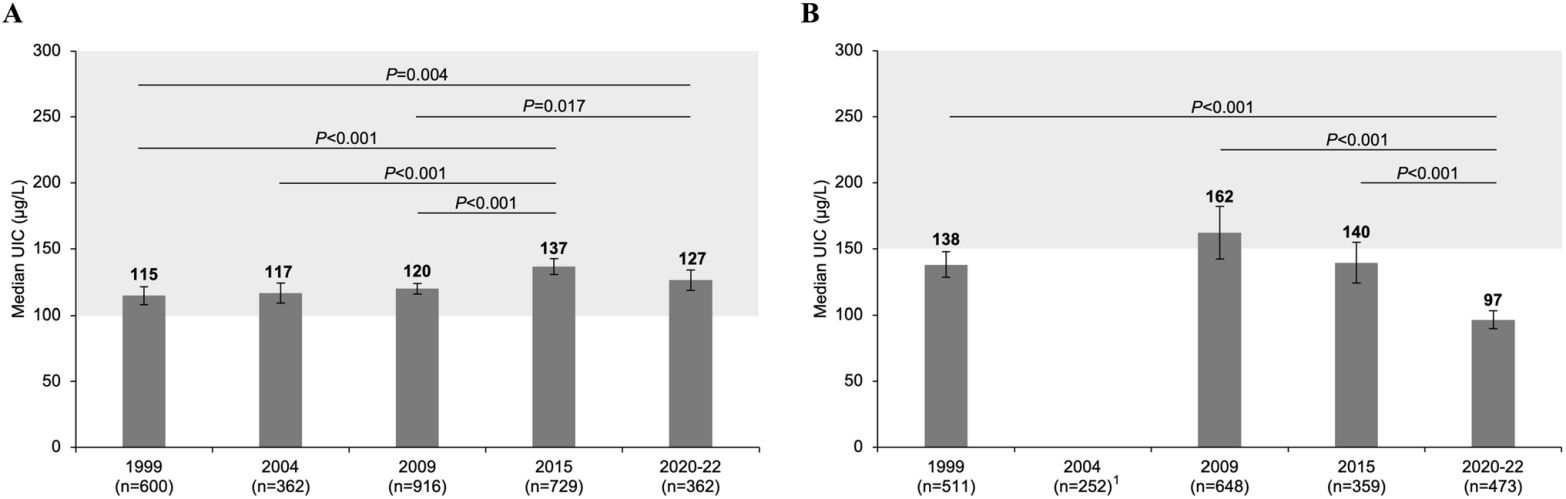
UIC in school-age children (**A**) and pregnant women (**B**) by year. Data are presented as median with 95% bootstrapped CIs. The shaded areas indicate adequate iodine nutrition according to WHO median UIC thresholds [8]. Kruskal-Wallis ANOVA followed by Mann-Whitney post hoc test with Bonferroni correction was used to test differences in median UIC between years. *P*-values are indicated for significant results only. ^1^Data excluded due to probable contamination with iodine. *n* sample size of the study, *UIC* spot urinary iodine concentration

Median UIC in children obtained from the first spot urine sample was 127 μg/L (bootstrapped 95% CI 119, 140, *n* = 362) and did not differ compared to the last national study in 2015 (137 µg/L, bootstrapped 95% CI 131, 143, n = 729; P = 0.682; Fig. 1A and Supplementary Table S2). The median UIC was ≥ 100 μg/L in all five of the geographic regions of Switzerland (Supplementary Table S3). When using the second urine sample to adjust for intra-individual variability, the median UIC increased to 143 μg/L (bootstrapped 95% CI 133, 153) (Table 2).

**Table 2:** Iodine, sodium and thyroid parameters of school-age children (*n* = 362) and pregnant women (*n* = 513) in the 2020–2022 Swiss national study

	School-age children		Pregnant women	
	Value^A^	*n*	Value	*n*
UIC (μg/L)^B^				
Sample 1	127 (119, 140) [IQR 87–194]^a^	362	97 (90, 106) [IQR 45–187]^C,a^	473
Sample 2	131 (118, 146) [IQR 86–172]^b^	96	107 (89, 125) [IQR 57–183]^a^	139
Adjusted UIC^D^	143 (133, 153)	338	123 (100, 145)	460
UCC (g/L)^B^				
Sample 1	0.87 (0.80, 0.94) [IQR 0.6–1.2]^a^	360	0.55 (0.48, 0.63) [IQR 0.3–1.0]^a^	472
Sample 2	0.93 (0.87, 1.08) [IQR 0.7–1.3]^b^	89	0.60 (0.51, 0.71) [IQR 0.4–1.0]^a^	138
Adjusted UCC^D^	0.95 (0.9, 1.0)	335	0.63 (0.5, 0.7)	460
UIC/UCC ratio (μg/g)^B^				
Sample 1	150 (144, 162) [IQR 106–217]^a^	360	166 (155, 183) [IQR 115–330]^a^	472
Sample 2	142 (122, 164) [IQR 102–195]^b^	85	161 (147, 185) [IQR 116–275]^a^	133
Adjusted estimated habitual iodine intake (μg/day)^D^	114 (102, 126)	330	269 (229, 307)^E^	457
No supplement users	114 (102, 126)	330	204 (178, 251)	232
Supplement users^F^	–	0	355 (316, 392)	195
Prevalence of inadequate iodine intake (%)^G^	5.4 (0.0, 14.6)	330	12.0 (3.2, 24.7)	457
No supplement users	5.4 (0.0, 14.6)	330	31.0 (10.5, 41.1)	232
Supplement users	–	0	0.2 (0.0, 4.0)	198
Prevalence of excessive iodine intake (%)^H^	0.0 (0.0, 0.2)	330	5.0 (0.0, 9.4)	457
No supplement users	0.0 (0.0, 0.2)	330	2.0 (0.0, 4.5)	232
Supplement users	–	0	3.4 (0.0, 10.7)	198
UNaC (mg/L)^B^				
Sample 1	3235 (3026, 3477) [IQR 2243–4476]^a^	359	–	–
Sample 2	3353 (2721, 3762) [IQR 2101–4411]^a^	96	–	–
Adjusted UNaC^D^	3342 (3184, 3513)	335	–	–
UNaC/UCC ratio (mg/g)^B^				
Sample 1	4049 (3721, 4388) [IQR 2463–5693]^I,a^	347	–	–
Sample 2	3288 (2930, 4104) [IQR 2026–5538]^b^	86	–	–
Adjusted estimated habitual sodium excretion (mg/day)^D^	2350 (2205, 2506)	319	–	–
Tg (μg/L)	–	–	26.0 (24.7, 28.4) [IQR 17.5–37.2]	465
Prevalence of elevated Tg (%)^J^	–	–	17.0	79
TSH (mU/L)	–	–	1.8 (1.8, 1.9) [IQR 1.6–2.0]	383
TT4 (nmol/L)	–	–	71.5 (69.1, 72.6) [IQR 59.5–81.7]	383
Prevalence of thyroid dysfunction (% [*n*])^K^	–	–	0.2 [1]^L^	383

Sodium was measured in school-age children only and thyroid function parameters in pregnant women only

*UIC* spot urinary iodine concentration, *UCC* spot urinary creatinine concentration, *UNaC* spot urinary sodium concentration, *Tg* thyroglobulin, *TSH* thyroid stimulating hormone, *TT4* total thyroxine

^A^Values are median (bootstrapped 95% CI) [interquartile range], all such values

^B^Wilcoxson signed-rank test was used to test differences between sample 1 and sample 2. Values with different superscript letters differed (*P *< 0.05)

^C^*n *= 40 outliers removed due to contamination with iodine

^D^Adjusted distribution accounting for intra-individual variability using the SPADE method and the package “SPADE-RIVM” [43] in R [36]

^E^*n* *=* 5 outliers removed (iodine intakes > 4000 μg/day)

^F^Supplement user defined as taking dietary supplements containing 150–220 μg iodine/day

^G^Percentage (bootstrapped 95% CI) of individuals with habitual intakes < AR of 65 μg/day for school-age children and 160 μg/day for pregnant women [42]

^H^Percentage of individuals with habitual intakes > UL of 450 μg/day for school-age children and 600 μg/day for pregnant women and from ESFA [45]. Applying the UL of 1100 μg/day for pregnant women from NAM, the prevalence of excessive iodine intake was 0% in supplement users and non-users, respectively [42]

^I^*n *= 7 outliers removed (sodium excretion > 15,000 mg/day)

^J^Defined as Tg > 43.5 μg/L [34]

^K^Defined as abnormal TSH and/or TT4 (including subclinical and overt hypothyroidism, subclinical and overt hyperthyroidism and isolated hypothyroxinaemia)

^L^One pregnant woman had subclinical hypothyroidism (defined as elevated TSH and normal TT4)

The median UCC was 0.86 g/L (bootstrapped 95% CI 0.80, 0.94) (Table 2). UIC was positively correlated with UCC (*r*_s_ = 0.498, *P* < 0.001) and UNaC (*r*_s_ = 0.393, *P* < 0.001), but did not correlate with age (*P* = 0.144). UCC correlated with UNaC (*r*_s_ = 0.267, *P* < 0.001) and age (*r*_s_ = 0.187, *P* < 0.001). UIC did not differ between girls and boys (*P* = 0.618), whereas UCC was higher in boys than in girls (*P* = 0.035). Median UIC did not differ depending on whether iodised salt was used in the household (87%) or not (13%) (*P* = 0.325). There was no difference in median UIC between children who abstained from eating certain foods (meat, fish, egg, milk, bread) compared to children who did not (*P* = 0.193). However, children who reported daily consumption of dairy products had significantly higher median UIC than children who did not (136 μg/L vs. 111 μg/L; *P* = 0.013). More specifically, in non-parametric testing we found that daily milk consumption was associated with UIC (*P* = 0.009), but daily yoghurt (*P* = 0.544), cheese (*P* = 0.585) and bread (*P* = 0.583) consumptions were not.

Multiple linear regression analysis was performed to determine the influence of log UCC, log UNaC, region and daily milk consumption on log UIC. Log UIC was predicted by log UCC (*P* < 0.001), log UNaC (*P* < 0.001), region (*P* = 0.002) and daily milk consumption (*P* < 0.001) (*F*(4,288) = 38.560, *P* < 0.001, *R*^2^ = 0.349; Supplementary Table S3). In multiple linear regression analysis including age, sex and region as independent variables, log UCC was influenced by age (*P* < 0.001) and sex (*P* = 0.010), but not by region (*P* = 0.151) (*F*(3,331) = 8.733, *P* < 0.001, *R*^2^ = 0.073).

#### Estimated habitual iodine intake and sodium excretion

The median habitual iodine intake in children was estimated at 114 μg/day (bootstrapped 95% CI 102, 126) by accounting for urine volume using UCC and adjusting for intra-individual variability using the second spot urine sample. The estimated prevalence of inadequate iodine intake (below the AR of 65 µg/day) was 5.4% (bootstrapped 95% CI 0.0, 14.6). None of the children (bootstrapped 95% CI 0.0, 0.2) had excessive intakes exceeding the UL of 450 µg/day (Table 2).

The median UNaC in children was 3235 mg/day (bootstrapped 95% CI 3026, 3477), with no difference compared to 2015 (Table 2 and Supplementary Table S2). The estimated median habitual sodium excretion was 2350 mg/day (bootstrapped 95% CI 2205, 2506) (Table 2). UNaC correlated with UIC (*r*_s_ = 0.393, *P* < 0.001), but the estimated individual iodine intake was not significantly associated with the estimated individual sodium excretion (*P* = 0.116).

#### Iodised salt

The salt iodine concentration was between 15 and 40 mg/kg in 81% of the collected household salt samples (*n* = 299). No iodine (< 5 mg/kg) was detected in 15% of the salt samples, but none of the samples had iodine concentrations above 40 mg/kg. The median iodine concentration in iodised salt samples (≥ 5 mg/kg) was 25.3 mg/kg (bootstrapped 95% CI 24.9, 25.6, *n* = 242). We observed no differences in the proportion of non-iodised salt samples or salt iodine concentrations between the Swiss geographical regions (*P* = 0.253). The iodine concentration in salt samples was positively correlated with the estimated individual iodine intake (*r*_s_ = 0.201, *P* < 0.001).

### Pregnant women

We enrolled 523 pregnant women from 22 obstetricians/gynaecologists and assessed their eligibility. We excluded 10 subjects as they did not fulfil the inclusion criteria and included 513 pregnant women in this study (Table 1). The age of the included women ranged from 18 to 44 years. The five Swiss regions were well represented, except for the Western region, where only 20% of the intended number of women were recruited. The North-eastern region was oversampled by 53% compared to the original protocol. Communities with a population < 10,000 inhabitants were underrepresented (63% of the indented sample size). A total of 76% of pregnant women (*n* = 352) had Swiss nationality and 25% (*n* = 112) were non-Swiss, reflecting the demographic structure in Switzerland [48]. Eighty-six per cent of the pregnant women reported using iodised salt in their homes. Seven per cent of the pregnant women reported thyroid disorders, of whom 5% suffered from hypothyroidism, all of whom were treated with thyroxine at the time of the study.

#### Urinary concentrations of iodine and creatinine

Median UIC in pregnant women measured in the first spot urine sample was 97 μg/L (bootstrapped 95% CI 90, 106, *n* = 473), 30% lower than the median of 140 μg/L (bootstrapped 95% CI 124, 159, n = 359) observed in 2015 (P = 0.004; Supplementary Table S2). When using the second urine sample to adjust for intra-individual variability, the median UIC increased to 123 μg/L (bootstrapped 95% CI 100, 145) (Table 2), but remained below the WHO threshold of 150 μg/L [8]. Over the years 1999 to 2020–2022, the median UIC fluctuated (*P* < 0.001) and declined steadily since 2009 (*P* < 0.001; post hoc analysis: Fig. 1B).  When using the second urine sample to adjust for intra-individual variability, the median UIC increased to 123 μg/L (bootstrapped 95% CI 100, 145) (Table 2), but remained below the WHO threshold of 150 μg/L [8].

**Fig. 2:**
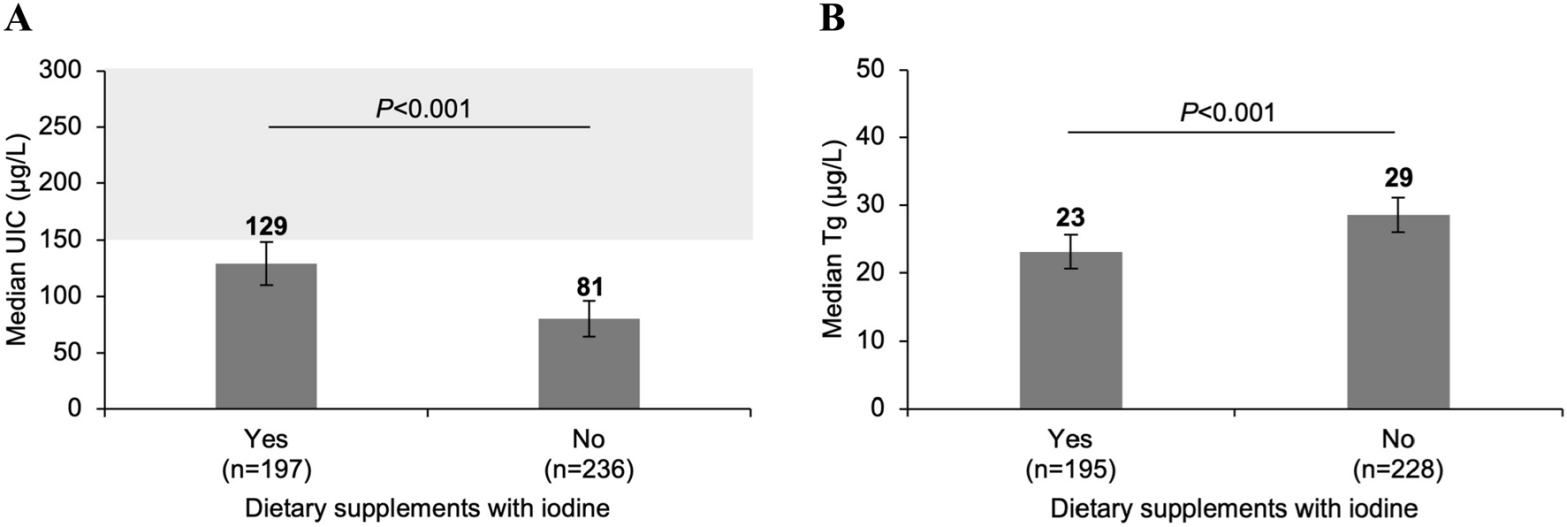
UIC (**A**) and Tg (**B**) of pregnant women with or without current consumption of dietary supplements containing 150–220 μg iodine/day. Data are presented as medians with 95% bootstrapped CIs. The shaded area indicates adequate iodine nutrition according to WHO median UIC thresholds [8]. Mann-Whitney *U* was used to test differences in median UIC/Tg. *n* sample size of the study, *UIC* spot urinary iodine concentration, *Tg* thyroglobulin

Almost half (47%) of the pregnant women consumed iodine-containing prenatal multivitamin and mineral supplements (containing 150–220 μg iodine/day) (Table 1), an increase compared to 2015 (37%, *P* = 0.039; Supplementary Table S2). The median UIC was higher in pregnant women consuming iodine-containing supplements compared to non-users (129 μg/L vs. 81 μg/L, *P* < 0.001; Fig. 2A and Supplementary Table S4).

The overall median UCC was low at 0.55 g/L (bootstrapped 95% CI 0.48, 0.63) (Table 2), suggesting a urine volume of approximately 2 L estimated using a daily UCE in Swiss non-pregnant women of 1.11 g/24 h [40]. UIC was positively correlated with UCC (*r*_s_ = 0.638, *P* < 0.001), but did not correlate with age (*P* = 0.131) or pre-pregnancy BMI (*P* = 0.088). UCC correlated negatively with age (*r*_s_ = − 0.111, *P* = 0.017) and positively with pre-pregnancy BMI (*r*_s_ = 0.122, *P* = 0.010). We observed no trimester differences in any of the urine parameters (UIC, *P* = 0.429; UCC, *P* = 0.063; Supplementary Table S4). Median UIC differed depending on educational level: women with a university degree had lower UIC (77 μg/L) compared to women who had completed an apprenticeship (120 μg/L) (post hoc analysis, *P* = 0.002). Similarly, median UCC of pregnant women with a university degree was lower (0.42 g/L), compared to women who had completed compulsory education (0.90 g/L) or an apprenticeship (0.65 g/L) (post hoc analysis, *P* = 0.002).

Median UIC was lower in pregnant women with Swiss nationality compared to those with a non-Swiss nationality (94 vs. 129 μg/L, *P* = 0.007) and the median UCC showed the same pattern (0.49 vs. 0.75 g/L, *P* < 0.001), suggesting more diluted urine in pregnant women with Swiss nationality. The median UIC and UCC in pregnant women differed between the regions of Switzerland (both *P* < 0.001; Supplementary Table S4). We observed no difference in the median UIC between women reporting consumption of iodized salt or those who did not (*P* = 0.054) or in women reporting daily consumption or abstaining from eating any of the iodine-containing food groups or not (fish: *P* = 0.500 and *P* = 0.641; bread: *P* = 0.790 and *P* = 0.957; dairy products: *P* = 0.676 and *P* = 0.752). Based on the Chi-square test iodine-containing supplement use differs by region (*P* < 0.001) as well as clinic (*P* < 0.001).

Multiple linear regression was performed to determine the influence of log UCC, iodine-containing supplement use, education, nationality and region on log UIC. Log UIC was associated with log UCC (*P* < 0.001), iodine-containing supplement use (*P* < 0.001) and region (*P* = 0.003) but not education (*P* = 0.064) or nationality (*P* = 0.230) (*F*(5,400) = 79.454, *P* < 0.001, *R*^2^ = 0.498; Supplementary Table S4).

A second multiple linear regression analysis was performed to determine the influence of age, pre-pregnancy BMI, education, nationality and region on log UCC. Log UCC was predicted by pre-pregnancy BMI (*P* = 0.019), education (*P* = 0.008) and nationality (*P* = 0.003), but not age (*P* = 0.096) and region (*P* = 0.296) (*F*(5,404) = 6.659, *P* < 0.001, *R*^2^ = 0.076; Supplementary Table S4).

#### Estimated habitual iodine intake

The estimated overall median habitual iodine intake was 269 μg/day (bootstrapped 95% CI 229, 307), accounting for urine volume using UCC and intra-individual variability using the second spot urine sample. The estimated prevalence of inadequacy, i.e. the percentage of individuals with usual intakes less than the AR of 160 μg/day, was 12.0% (bootstrapped 95% CI 3.2, 24.7). The estimated habitual iodine intake in pregnant women consuming iodine-containing dietary supplements was higher than that of non-users (355 vs. 204 μg/day, *P* < 0.001; Table 2). The prevalence of inadequate iodine intake was 0.2% (bootstrapped 95% CI 0.0, 4.0) in women consuming iodine-containing supplements, compared to 31.0% (bootstrapped 95% CI 10.5, 41.1) in non-users. The prevalence of excessive iodine intake, i.e. percentages of individuals with usual intakes exceeding the EFSA UL of 600 μg/day, was 3.4% (bootstrapped 95% CI 0.0, 10.7) in pregnant women consuming iodine-containing supplements, compared to 2.0% (bootstrapped 95% CI 0.0, 4.5) in non-users. However, when applying the UL of 1100 μg/day by the NAM, the prevalence of excessive iodine intake was 0% in both groups.

#### Thyroid function parameters

The median DBS-Tg concentration in pregnant women was 26.0 μg/L (bootstrapped 95% CI 24.7, 28.4) and increased compared to 2015 (23.8 μg/L, bootstrapped 95% CI 22.1, 26.1, *P* = 0.014). However, the prevalence of elevated DBS-Tg (> 43.5 μg/L) did not differ compared to 2015 (17% vs 13%, *P* = 0.145). The median Tg concentration and the prevalence of elevated Tg were lower in women taking iodine-containing supplements, compared to non-users (Fig. 2B and Supplementary Table S4). We found an overall difference in median DBS-Tg concentration between the regions of Switzerland (*P* < 0.001; Supplementary Table S4). Multiple linear regression was performed to determine the overall influence of age, iodine-containing supplement use, education, nationality and region on log Tg (*F*(5,368) = 4.975, *P* < 0.001, *R*^2^ = 0.063): age (*P* = 0.018) and the consumption of iodine-containing supplements (*P* < 0.001) predicted log Tg, but education (*P* = 0.226), nationality (*P* = 0.883) and region (*P* = 0.102) did not.

The median DBS-TSH and DBS-TT4 concentrations represent an overall euthyroid population (Table 2). Only one participant had subclinical hypothyroidism (0.2%), while all other pregnant women had normal thyroid function. We found no difference in median DBS-TSH and DBS-TT4 between women consuming iodine-containing supplements and those who did not (TSH, *P* = 0.060; TT4, *P* = 0.065). We observed no trimester differences in the median Tg (*P* = 0.897) and TSH (*P* = 0.133), but as expected TT4 was lower in the first trimester compared to the third (*P* = 0.013). We found no correlation between UIC and Tg, TSH or TT4 (*P* = 0.117; *P* = 0.146 and *P* = 0.983, respectively).

## Discussion

This nationwide cross-sectional study confirms adequate iodine intake in Swiss school-age children and suggests that one-third of pregnant women do not get enough iodine from the diet alone. However, approximately half of pregnant women in Switzerland consume an iodine-containing dietary supplement every day and this helps to ensure iodine adequacy.

In trend analysis, we show that the median UIC in children remained relatively stable over the years 1999–2022, although still at the lower end of the adequate range. A modest improvement in median UIC was observed in 2015 after increasing the salt iodine level from 20 to 25 mg/kg in 2014 [3]. This increase was maintained in the current study and the proportion of children with inadequate habitual iodine intake decreased from 10 to 5% (Supplementary Table S2). At the same time, our data also suggest a decline in iodine intake in pregnant women since 2015, indicated by lower median UIC and higher median Tg, especially in women not consuming iodine-containing supplements (Fig. 2).

The origin of iodine from different dietary sources cannot directly be assessed in this study and the contribution of salt to the total iodine intake can only be estimated. Studies in other countries show that mandatory salt iodization at 25 mg/kg ensures adequate dietary iodine intake in all population groups, including pregnant women with increased requirements [8, 49]. However, we found an elevated prevalence of low intakes in pregnant women suggesting that the iodised salt coverage may be insufficient. We confirm that the use of iodised salt by households remains high (> 85%), but the coverage in processed foods is incomplete. A recent market survey indicated that only one-third of processed foods are produced using iodised salt: 47% of foods manufactured for the local market contain iodised salt, but only 9% of imported products [4]. We observed no difference in the UNaC concentration in children compared to 2015 [3] (Supplementary Table S2), despite national and international recommendations to reduce the salt intake [50, 51]. The estimated sodium excretion of 2.4 g/day (5.9 g salt/day) is still well above the national [52] and international recommendations [53]. Our result agrees with a recent local study in children [54] and nationally representative data in adults [55]. UNaC predicted UIC in children in our study population, although moderately. Salt sales data show that 60% of edible salt sold in Switzerland is iodised (Personal communication, Swiss Saltworks 2021). Consequently, children may consume approximately 90 µg iodine per day from salt (5.9 g salt/day × 60% iodised × 25 mg/kg), making salt the main dietary source of iodine, but it does not alone meet the dietary requirements. As in many other European countries [56], milk and dairy products are also important dietary sources of iodine in the Swiss population [14, 57], especially in children [58]. In our study, children who consumed dairy products daily had higher UIC than those who abstained. A high proportion (> 50%) of the study population also consumed bread on a daily basis and according to a recent survey 86% of bakeries use iodised salt [59].

Motivating the food industry to use iodised salt in selected food products that are frequently consumed may be the most effective way to increase iodine intake. Information campaigns targeted to food producers and the general public may also be useful. The level of iodine fortification could be further increased, but must likely be higher than the previous increase of 5 mg/kg to be efficacious [3].

Our results show that iodine supplementation during pregnancy improves the iodine intake. Iodine supplementation is not explicitly recommended in Switzerland, but women taking a prenatal multivitamin and mineral supplement on a regular basis (96%, Table 1) are advised to choose a product that contains iodine [60]. The consumption of iodine-containing supplements during pregnancy increased from 15% in 2009 to 41% in 2015 [3] and to 47% in 2020–2022. Iodine (≥ 150 μg) is included in 61% of the prenatal supplements available on the Swiss market [61], but the prenatal product most frequently (38%) prescribed to women in our study did not contain iodine. Physicians and pharmacists providing supplement products need to be aware about the variability in iodine content of available supplements [61]. The clinical benefits of iodine supplementation in pregnant women with mild iodine deficiency remain uncertain [62, 63]. In our study, all women were euthyroid and the thyroid function was unaffected also in women not taking supplements. Further, supplementation with multivitamin and mineral products is generally started at the end of the first trimester and may miss the critical period of rapid thyroid and brain development that occurs during early pregnancy [3]. Thus, optimisation of iodine intake should start before pregnancy, such that intrathyroidal iodine stores are optimised before conception [64]. Our data show that the risk of iodine excess from iodine supplements is low. The prevalence of excessive iodine intake was 3% in supplement users when applying the UL by ESFA (> 600 μg/day [45]). High iodine intakes are generally well tolerated in healthy individuals [65] and the prevalence reduced to 0% when applying the UL of NAM (1100 μg/day [42]).

Our study also exemplifies a few limitations in the way iodine adequacy is currently defined. The WHO UIC cut-offs are based on the association between the estimated daily iodine excretion and the risk of goitre in the population [8]. Epidemiological studies suggest that an average daily iodine excretion lower than 100 µg increases the goitre prevalence among children and adults [66–68]. However, the WHO guidelines converted the iodine excretion into a corresponding urine concentration, using a given urine volume [8]. In children, a daily excretion of 100 µg/day equals to a UIC of 100 µg/L if the 24 h urine volume is equivalent to 1 L [8]. However, if the urine volume is lower than 1 L, as suggested in our study (UCE reference of 0.57 g/day [39]/UCC of 0.95 g/L = 0.6 L) and other studies [69], the WHO median UIC threshold (< 100 µg/L) may mask iodine inadequacy. During pregnancy, the daily dietary iodine requirement is 250 µg/day [70] and the WHO median UIC cut-off for adequacy (≥ 150 µg/L) assumes a urine volume of 1.5 L [8]. We estimated a 30% larger urine volume in pregnant women (UCE reference of 1.11 g/day [40]/UCC of 0.55 g/L = 2L), in agreement with earlier studies collecting 24 h urine in Swiss adults [9, 14, 28]. In women consuming a dietary iodine supplement almost all women had adequate iodine intake even if the median UIC was below 150 µg/L (Fig. 2A; Table 2), suggesting that this WHO median UIC cut-off may overestimate iodine deficiency in populations with high urine volume.

Our study evaluated several possibilities to improve the interpretation of population iodine status in UIC studies. First, we collected a repeat spot urine sample and adjusted the UIC concentration for intra-individual variability [10, 27]. The high variability in the iodine intake is well recognised [10, 27]. Due to the skewness in the data, the adjusted median UIC increased compared to the crude median UIC and likely provided a more reliable estimate of the habitual UIC. In our study setting, a repeat urine sample was easily obtained by asking the participants to send the second urine sample to the laboratory by mail. Second, we calculated the individual iodine intake from UIC by accounting for the urine dilution using the UCC measured in spot urine (Eq. 1a, 1b), assuming a constant urinary creatinine excretion over 24 h [38]. In pregnant women, UCE reference values are lacking and the use of age- and sex-specific creatinine values based on non-pregnant Swiss women [40] is possibly a limitation as the UCE may be altered in pregnancy [71]. UCC improves the interpretation of the iodine intake compared to UIC alone, as discussed above. Third, we estimated the habitual iodine intake distribution using the repeat urine sample and applied the AR cut-point method to estimate the prevalence of iodine inadequacy [10, 46]. We found that approximately 12% of all pregnant women had intakes below the requirements even if the overall median habitual iodine intake appeared adequate, and this prevalence agrees well with previous studies in Swiss adults [9, 14, 28]. The additional information of the prevalence of inadequate iodine intake identified disparities in the habitual iodine intakes and is more informative than the median UIC alone.

We recognise the poor response rate of schools and obstetric clinics in our study and acknowledge that a cluster-based study design as recommended by WHO [8] is becoming more and more difficult to implement, particularly in schools. We obtained a demographically balanced sample of pregnant women and a nationwide sample of children, but cannot confirm that they are truly nationally representative. We also recognise that the small sample size in children may impact on the precision and present the bootstrapped 95% CI around the median for all parameters to indicate the uncertainty. Despite strict sample collection instructions, we found unexplained high UIC in urine samples collected in pregnant women from three clinics. This finding suggested potential iodine contamination from glucose test strips and we removed the data from the analysis. We cannot exclude iodine contamination in a few individual samples from other clinics. Potential sporadic iodine contamination affects the median UIC minimally but may shift the adjusted UIC and intake distributions towards higher intakes.

To conclude, we show that the iodine intake in Switzerland is sufficient in children. However, the intake distribution is marginally low in pregnant women, particularly in those not consuming iodine supplements. Although the household coverage of iodized salt remains high, improved use in processed foods would be desired to prevent iodine inadequacy in all population groups and avoid exposure to iodine deficiency in early pregnancy.

### Supplementary Information

Below is the link to the electronic supplementary material.

Supplementary file1 (DOCX 63 kb)

## Data Availability

Data described in the manuscript, code book, and analytic code will be made available upon request in a deidentified form pending application and approval.

## Abbreviations

AR: Average requirement
DBS: Dried blood spot
EFSA: European Food Safety Authority
NAM: US National Academy of Medicine (previously US Institute of Medicine, IOM)
Tg: Thyroglobulin
TSH: Thyroid stimulating hormone
TT4: Total thyroxine
UCC: Urinary creatinine concentration
UIC: Urinary iodine concentration
UIE: Urinary iodine excretion
UNaC: Urinary sodium concentration
UL: Upper level

## References

[1] Bürgi H, Supersaxo Z, Selz B (1990). Iodine deficiency diseases in Switzerland one hundred years after Theodor Kocher's survey: a historical review with some new goitre prevalence data. Acta Endocrinol (Copenh).

[2] Zimmermann M, Andersson M (2021). Global endocrinology: global perspectives in endocrinology: coverage of iodized salt programs and iodine status in 2020. Eur J Endocrinol.

[3] Andersson M, Hunziker S, Fingerhut R, Zimmermann M, Herter-Aeberli I (2019). Effectiveness of increased salt iodine concentration on iodine status: trend analysis of cross-sectional national studies in Switzerland. Eur J Clin Nutr.

[4] FDHA (2022) Verwendung von jodiertem Salz in industriell verarbeiteten Lebensmitteln: Markterhebung. Risikobewertung. Federal Food Safety and Veterinary Office (FSVO), Bern

[5] Andersson M, Aeberli I, Wüst N, Piacenza A, Bucher T, Henschen I, Haldimann M, Zimmermann M (2010). The Swiss iodized salt program provides adequate iodine for school children and pregnant women, but weaning infants not receiving iodine-containing complementary foods as well as their mothers are iodine deficient. J Clin Endocrinol Metab.

[6] Hess S, Zimmermann M, Torresani T, Bürgi H, Hurrell R (2001). Monitoring the adequacy of salt iodization in Switzerland: a national study of school children and pregnant women. Eur J Clin Nutr.

[7] Zimmermann M, Aeberli I, Torresani T, Bürgi H (2005). Increasing the iodine concentration in the Swiss iodized salt program markedly improved iodine status in pregnant women and children: a 5-y prospective national study. Am J Clin Nutr.

[8] WHO (2007). Assessment of iodine deficiency disorders and monitoring their elimination: a guide for programme managers.

[9] Stalder E, Haldimann M, Blanc A, Dudler V, Ponte B, Pruijm M, Ackermann D, Bochud M (2019). Use of day and night urinary iodine excretion to estimate the prevalence of inadequate iodine intakes via the estimated average requirement cut-point method. Swiss Med Wkly.

[10] Zimmermann M, Andersson M (2012). Assessment of iodine nutrition in populations: past, present, and future. Nutr Rev.

[11] Dorey C, Zimmermann M (2008). Reference values for spot urinary iodine concentrations in iodine-sufficient newborns using a new pad collection method. Thyroid.

[12] Ittermann T, Albrecht D, Arohonka P, Bilek R, de Castro J, Dahl L, Filipsson Nystrom H, Gaberscek S, Garcia-Fuentes E, Gheorghiu M, Hubalewska-Dydejczyk A, Hunziker S, Jukic T, Karanfilski B, Koskinen S, Kusic Z, Majstorov V, Makris K, Markou K, Meisinger C, Milevska Kostova N, Mullen K, Nagy E, Pirags V, Rojo-Martinez G, Samardzic M, Saranac L, Strele I, Thamm M, Top I, Trofimiuk-Müldner M, Ünal B, Koskinen S, Vila L, Vitti P, Winter B, Woodside J, Zaletel K, Zamrazil V, Zimmermann M, Erlund I, Völzke H (2020). Standardized map of iodine status in Europe. Thyroid.

[13] EDI (2022) Verordnung des EDI über den Zusatz von Vitaminen, Mineralstoffen und sonstigen Stoffen in Lebensmitteln (VZVM) (817.022.32). vom 16. Dezember 2016 (Stand am 1. Juli 2022). Federal Department of Home Affairs (FDHA), Bern

[14] Haldimann M, Bochud M, Burnier M, Paccaud F, Dudler V (2014). Prevalence of iodine inadequacy in Switzerland assessed by the estimated average requirement cut-point method in relation to the impact of iodized salt. Public Health Nutr.

[15] Benzi-Schmid C, Haldimann M (2019) Sind Milch und Milchprodukte gute Jodquellen? Schweizer Ernährungsbulletin 2019. BLV, Bundesamt für Lebensmittelsicherheit und Veterinärwesen, Bern. doi:10.24444/blv-2018-0111

[16] Benzi-Schmid C (2023) Nahrungsmittelbilanz für die Schweiz: Überblick zum angenäherten Verzehr und zu dessen Entwicklung in den vergangenen acht Jahren. Schweizer Ernährungsbulletin. Federal Food Safety and Veterinary Office (FSVO), Bern, Switzerland. 10.24444/blv-2023-0111

[17] Agristat S, Switzerland Cheese Marketing, TSM Treuhand GMbH, Branchenorganisation Milch (2022) Dairy statistics Switzerland 2021. Branchenorganisation Milch, Brugg

[18] Chatelan A, Beer-Borst S, Randriamiharisoa A, Pasquier J, Blanco J, Siegenthaler S, Paccaud F, Slimani N, Nicolas G, Camenzind-Frey E, Zuberbuehler C, Bochud M (2017). Major differences in diet across three linguistic regions of Switzerland: results from the first national nutrition survey menuCH. Nutrients.

[19] Bochud M, Chatelan A, Blanco J, Beer-Borst S (2017) Anthropometric characteristics and indicators of eating and physical activity behaviors in the Swiss adult population: Results from menuCH 2014/2015. Federal Office of Public Health and the Food Safety and Veterinary Office. 10.7892/boris.101641

[20] FDHA (2021) Schweizer Ernährungsbulletin 2021. Federal Food Safety and Veterinary Office (FSVO), Bern

[21] FCN (2018) Vegan diets: review of nutritional benefits and risks. Expert report of the FCN. Federal Food Savety and Veterinary Office, Bern

[22] Swissveg (2022) Statistics on vegetarians and vegans in Switzerland. Swissveg, Winterthur

[23] Schüpbach R, Wegmüller R, Berguerand C, Bui M, Herter-Aeberli I (2017). Micronutrient status and intake in omnivores, vegetarians and vegans in Switzerland. Eur J Nutr.

[24] Walther B, Guggisberg D, Badertscher R, Egger L, Portmann R, Dubois S, Haldimann M, Kopf-Bolanz K, Rhyn P, Zoller O, Veraguth R, Rezzi S (2022). Comparison of nutritional composition between plant-based drinks and cow's milk. Front Nutr.

[25] FSO (2021) Population and households statistics (STATPOP) 2020. Bern

[26] Fraser C, Harris E (1989). Generation and application of data on biological variation in clinical chemistry. Crit Rev Clin Lab Sci.

[27] König F, Andersson M, Hotz K, Aeberli I, Zimmermann M (2011). Ten repeat collections for urinary iodine from spot samples or 24-hour samples are needed to reliably estimate individual iodine status in women. J Nutr.

[28] Arns-Glaser L, Zihlmann R, Gessler S, Verkaik-Kloosterman J, Zandberg L, Assey V, Rigutto-Farebrother J, Braegger C, Zimmermann M, Andersson M (2023). Estimating habitual iodine intake and prevalence of inadequacy from spot urine in cross-sectional studies: a modeling analysis to determine the required sample size. Am J Clin Nutr.

[29] Lachat C, Hawwash D, Ocké M, Berg C, Forsum E, Hörnell A, Larsson C, Sonestedt E, Wirfält E, Åkesson A, Kolsteren P, Byrnes G, De Keyzer W, Van Camp J, Cade J, Slimani N, Cevallos M, Egger M, Huybrechts I (2016). STrengthening the Reporting of OBservational studies in Epidemiology—nutritional epidemiology (STROBE-nut): an extension of the STROBE statement. PLoS Med.

[30] WHO (1995). Physical status: the use and interpretation of anthropometry—report of a WHO expert committee.

[31] Pino S, Fang S, Braverman L (1996). Ammonium persulfate: a safe alternative oxidizing reagent for measuring urinary iodine. Clin Chem.

[32] Vasiliades J (1976). Reaction of alkaline sodium picrate with creatinine: kinetics and mechanism of formation of the mono-creatinine picric acid complex. Clin Chem.

[33] Stinca S, Andersson M, Erhardt J, Zimmermann M (2015). Development and validation of a new low-cost enzyme-linked immunoassay for serum and dried blood spot thyroglobulin. Thyroid.

[34] Stinca S, Andersson M, Weibel S, Herter-Aeberli I, Fingerhut R, Gowachirapant S, Hess S, Jaiswal N, Jukic T, Kusic Z, Mabapa N, Nepal A, San Luis T, Zhen J, Zimmermann M (2017). Dried blood spot thyroglobulin as a biomarker of iodine status in pregnant women. J Clin Endocrinol Metab.

[35] Alexander E, Pearce E, Brent G, Brown R, Chen H, Dosiou C, Grobman W, Laurberg P, Lazarus J, Mandel S, Peeters R, Sullivan S (2017). 2017 Guidelines of the American Thyroid Association for the diagnosis and management of thyroid disease during pregnancy and the postpartum. Thyroid.

[36] R Development Core Team (2021) R: a language and environment for statistical computing. 4.1.2. edn. R Foundation for Statistical Computing, Vienna

[37] Pearce E, Lazarus J, Smyth P, He X, Smith D, Pino S, Braverman L (2009). Urine test strips as a source of iodine contamination. Thyroid.

[38] Montenegro-Bethancourt G, Johner S, Stehle P, Neubert A, Remer T (2015). Iodine status assessment in children: spot urine iodine concentration reasonably reflects true twenty-four-hour iodine excretion only when scaled to creatinine. Thyroid.

[39] Remer T, Neubert A, Maser-Gluth C (2002). Anthropometry-based reference values for 24-h urinary creatinine excretion during growth and their use in endocrine and nutritional research. Am J Clin Nutr.

[40] Forni Ogna V, Ogna A, Vuistiner P, Pruijm M, Ponte B, Ackermann D, Gabutti L, Vakilzadeh N, Mohaupt M, Martin P, Guessous I, Péchère-Bertschi A, Paccaud F, Bochud M, Burnier M (2015). New anthropometry-based age- and sex-specific reference values for urinary 24-hour creatinine excretion based on the adult Swiss population. BMC Med.

[41] Jahreis G, Hausmann W, Kiessling G, Franke K, Leiterer M (2001). Bioavailability of iodine from normal diets rich in dairy products—results of balance studies in women. Exp Clin Endocrinol Diabetes.

[42] IOM (2001) Dietary reference intakes for vitamin A, vitamin K, arsenic, boron, chromium, copper, iodine, iron, manganese, molybdenum, nickel, silicon, vanadium, and zinc, vol Iodine, vol 8. National Academies Press (US), Washington (DC) 25057538

[43] Dekkers A, Verkaik-Kloosterman J, van Rossum C, Ocké M (2014). SPADE, a new statistical program to estimate habitual dietary intake from multiple food sources and dietary supplements. J Nutr.

[44] Allen L, Carriquiry A, Murphy S (2020). Perspective: proposed harmonized nutrient reference values for populations. Adv Nutr.

[45] EFSA (2018) Summary of tolerable upper intake levels—version 4 (September 2018). European Food Safety Authority: Scientific Committee on Food and Scientific Panel on Dietetic Products, Nutrition and Allergies

[46] IOM (2000) DRI dietary reference intakes: applications in dietary assessment. National Academies Press (US), Washington (DC). 10.17226/9956 25057725

[47] Murphy S, Yaktine A, Carriquiry A (2021). Planning nutritionally adequate diets for groups: methods used to develop recommendations for a child and adult care food program. Adv Nutr.

[48] FSO (2021) Population by migration status 2021. https://www.bfs.admin.ch/bfs/en/home/statistics/population/migration-integration/by-migration-status.html. Accessed 05 May 2023

[49] Dold S, Zimmermann M, Jukic T, Kusic Z, Jia Q, Sang Z, Quirino A, San Luis T, Fingerhut R, Kupka R, Timmer A, Garrett G, Andersson M (2018). Universal salt iodization provides sufficient dietary iodine to achieve adequate iodine nutrition during the first 1000 days: a cross-sectional multicenter study. J Nutr.

[50] FDHA (2017) Swiss nutrition policy 2017–2024. Federal Food Safety and Veterinary Office (FSVO), Bern, Switzerland

[51] WHO (2014). Salt reduction and iodine fortification strategies in public health: report of a joint technical meeting.

[52] Strohm D, Bechthold A, Ellinger S, Leschik-Bonnet E, Stehle P, Heseker H (2018). Revised reference values for the intake of sodium and chloride. Ann Nutr Metab.

[53] WHO (2012). Guideline: sodium intake for adults and children.

[54] Rios-Leyvraz M, Bovet P, Bochud M, Genin B, Russo M, Rossier M, Tabin R, Chiolero A (2019). Estimation of salt intake and excretion in children in one region of Switzerland: a cross-sectional study. Eur J Nutr.

[55] Chappuis A, Bochud M, Glatz N, Vuistiner P, Paccaud F, Burnier M (2011) Swiss survey on salt intake: main results. Federal Office of Public Health

[56] Bath S, Verkaik-Kloosterman J, Sabatier M, Ter Borg S, Eilander A, Hora K, Aksoy B, Hristozova N, van Lieshout L, Tanju Besler H, Lazarus J (2022). A systematic review of iodine intake in children, adults, and pregnant women in Europe—comparison against dietary recommendations and evaluation of dietary iodine sources. Nutr Rev.

[57] Haldimann M, Alt A, Blanc A, Blondeau K (2005). Iodine content of food groups. J Food Compost Anal.

[58] van der Reijden O, Zimmermann M, Galetti V (2017). Iodine in dairy milk: sources, concentrations and importance to human health. Best Pract Res Clin Endocrinol Metab.

[59] Stalder U, Haldimann M (2021) Salz im Brot—darf es eine Prise weniger sein? Bundesamt für Lebensmittelsicherheit und Veterinärwesen (BLV), Bern. 10.24444/blv-2021-0111

[60] FDHA (2019) Ernährung in Schwangerschaft und Stillzeit. Federal Food Safety and Veterinary Office (FSVO), Bern, Switzerland

[61] Gfeller M, Colque G, Kopp P (2022). Iodine content of frequently used prenatal and adult multivitamins in Switzerland. Front Endocrinol.

[62] Zimmermann M (2020). Iodine supplements for mildly iodine-deficient pregnant women: are they worthwhile?. Am J Clin Nutr.

[63] Dineva M, Fishpool H, Rayman M, Mendis J, Bath S (2020). Systematic review and meta-analysis of the effects of iodine supplementation on thyroid function and child neurodevelopment in mildly-to-moderately iodine-deficient pregnant women. Am J Clin Nutr.

[64] Næss S, Markhus M, Strand T, Kjellevold M, Dahl L, Stokland A, Nedrebø B, Aakre I (2021). Iodine nutrition and iodine supplement initiation in association with thyroid function in mildly-to-moderately iodine-deficient pregnant and postpartum women. J Nutr.

[65] Farebrother J, Zimmermann M, Andersson M (2019). Excess iodine intake: sources, assessment, and effects on thyroid function. Ann NY Acad Sci.

[66] Ascoli W, Arroyave G (1970). Epidemiologia el bocio endemico en Centro America. Relacion entre prevalencia y excrecion urinaria de yodo (Epidemiology of endemic goiter in Central America. Association between prevalence and urinary iodine excretion). Arch Latinoamer Nutr.

[67] Langer P, Stanbury Hbe JB (1980). Eastern and southeastern Europe. Endemic goiter and endemic cretinism.

[68] Delange F, Benker G, Caron P, Eber O, Ott W, Peter F, Podoba J, Simescu M, Szybinsky Z, Vertongen F, Vitti P, Wiersinga W, Zamrazil V (1997). Thyroid volume and urinary iodine in European schoolchildren: standardization of values for assessment of iodine deficiency. Eur J Endocrinol.

[69] Beckford K, Grimes C, Margerison C, Riddell L, Skeaff S, West M, Nowson C (2020). A systematic review and meta-analysis of 24h urinary output of children and adolescents: impact on the assessment of iodine status using urinary biomarkers. Eur J Nutr.

[70] FDHA (2018) Empfehlungen zu Jod. Federal Food Safety and Veterinary Office (FSVO), Bern

[71] Bu Y, Yuan L, Tian C, Zhao C, Ji C, Gao X, Cai Y, Sun D, Liu Y (2021). 24h urinary creatinine excretion during pregnancy and its application in appropriate estimation of 24h urinary iodine excretion. J Trace Elem Med Biol.

