# Correction to: Iodine intake in the Swiss population 100 years after the introduction of iodised salt: a cross-sectional national study in children and pregnant women

**DOI:** 10.1007/s00394-024-03331-z

**Published:** 2024-02-28

**Authors:** Lena Fischer, Maria Andersson, Christian Braegger, Isabelle Herter-Aeberli, Swiss Iodine Study Collaborators

**Affiliations:** 1 Nutrition Research Unit, Children’s Research Centre, University Children’s Hospital Zurich - Eleonore Foundation, Steinwiesstrasse 75, 8032 Zurich, Switzerland; 2 Laboratory of Nutrition and Metabolic Epigenetics, Institute of Food, Nutrition and Health, ETH Zurich, Zurich, Switzerland

**Correction to** **:** **European Journal of Nutrition** 10.1007/s00394-023-03287-6

In the original version of this article, Swiss Iodine Study Collaborators was missing in the author groups. Author groups should have read:

Lena Fischer^1,2^ · Maria Andersson^1^ · Christian Braegger^1^ · Isabelle Herter-Aeberli^2^ · Swiss Iodine Study Collaborators

The original article has been corrected.

