# Supplementary material for: Iodine intake in the Swiss population 100 years after the introduction of iodised salt: a cross-sectional national study in children and pregnant women
Source: Eur J Nutr. 2023 Dec 23;63(2):573–87. doi: 10.1007/s00394-023-03287-6 (PMC10899291; doi:10.1007/s00394-023-03287-6)
Supplement: Supplementary file 1 — Supplementary file1 (DOCX 63 kb) [file 394_2023_3287_MOESM1_ESM.docx]

**Online Supplementary Material**

**Title:**

Iodine intake in the Swiss population 100 years after the introduction of iodised salt: A cross-sectional national study in children and pregnant women

**Journal name:**

European Journal of Nutrition

**Authors:**

Lena Fischer*****, Maria Andersson, Christian Braegger, Isabelle Herter-Aeberli

*Corresponding author: [lena.fischer@kispi.uzh.ch](mailto:lena.fischer@kispi.uzh.ch)

Nutrition Research Unit, Children’s Research Centre, University Children’s Hospital Zurich, Zurich, Switzerland

Laboratory of Nutrition and Metabolic Epigenetics, Institute of Food, Nutrition and Health, ETH Zurich, Zurich, Switzerland

**Contents:**

**Supplementary Table S1** External quality control for the laboratory analysis

**Supplementary Table S2** Iodine, sodium and thyroid parameters of school-age children and pregnant women in the 2020-2022 and 2015 Swiss national study

**Supplementary Table S3** Determinants of urinary iodine and creatinine concentrations in school-age children (*n=*362) in the 2020-2022 Swiss national study

**Supplementary Table S4** Determinants of urinary iodine concentration, urinary creatinine concentration and thyroglobulin in pregnant women (*n=*513) in the 2020-2022 Swiss national study

**Supplementary Table S1** External quality control for the laboratory analysis

| Parameter | Inter-assay variability | | | | | | Reference material |
| --- | --- | --- | --- | --- | --- | --- | --- |
|  | Mean (µg/L) | Coefficient of variation (%) | *n* | Mean (µg/L) | Coefficient of variation (%) | *n* |  |
| UIC | 59 | 7 | 47 | 186 | 3 | 47 | External quality control urine samples provided by ETH Zurich |
| UCC | 99 | 6 | 48 | 205 | 4 | 48 | External quality control urine samples provided by ETH Zurich |
| UNaC | 1.7^1^ | 5 | 12 | 4.2^1^ | 2 | 12 | Seronorm Trace Elements Urine Levels; Sero, Norway |
| SIC^2^ | 61 | 5 | 17 | 188 | 3 | 17 | External quality control urine samples provided by ETH Zurich |
| Tg | 23 | 21 | 14 | 46 | 11 | 14 | Liquicheck Tumor Marker Control; Bio-Rad, Hercules, CA, USA |

UIC, urinary iodine concentration; UCC*,* urinary creatinine concentration; UNaC*,* urinary sodium concentration; SIC, salt iodine concentration; Tg*,* thyroglobulin.

^1^Unit in g/L; ^2^Urine sample used as quality control.

**Supplementary Table S2** Iodine, sodium and thyroid parameters of school-age children and pregnant women in the 2020-2022 and 2015 Swiss national study

|  | School-age children |  |  |  |  |  | Pregnant women |  |  |  |  |
| --- | --- | --- | --- | --- | --- | --- | --- | --- | --- | --- | --- |
|  | 2020-2022  (*n=*362) |  |  | 2015 [1]  (*n=*731) |  |  | 2020-2022  (*n=*513) |  |  | 2015 [1]  (*n=*363) |  |
|  | Value | *n* |  | Value | *n* |  | Value | *n* |  | Value | *n* |
| UIC (μg/L)^1,2^ |  |  |  |  |  |  |  |  |  |  |  |
| Sample 1 | 127 (87–194)^a^ | 362 |  | 137 (100–188)^a^ | 729 |  | 97 (45–187)^3,a^ | 473 |  | 140 (65–314)^a^ | 359 |
| Sample 2 | 131 (86–172)^b^ | 96 |  | 145 (102–192)^a^ | 202 |  | 107 (57–183)^a^ | 139 |  | 97 (40–164)^b^ | 102 |
| Adjusted UIC^4,5^ | 143 (133, 153) | 338 |  | 148 (142, 153) | 684 |  | 123 (100, 145) | 460 |  | 206 (170, 245) | 348 |
| UCC (g/L)^1,2^ |  |  |  |  |  |  |  |  |  |  |  |
| Sample 1 | 0.87 (0.6–1.2)^a^ | 360 |  | 1.05 (0.8–1.3)^a^ | 709 |  | 0.55 (0.3–1.0)^a^ | 472 |  | – | – |
| Sample 2 | 0.93 (0.7–1.3)^b^ | 89 |  | 1.01 (0.8–1.3)^a^ | 198 |  | 0.60 (0.4–1.0)^a^ | 138 |  | – | – |
| Adjusted UCC^4,5^ | 0.95 (0.9, 1.0) | 335 |  | 1.07 (1.0, 1.1) | 669 |  | 0.63 (0.5, 0.7) | 460 |  | – | – |
| UIC/UCC ratio (μg/g)^1,2^ |  |  |  |  |  |  |  |  |  |  |  |
| Sample 1 | 150 (106–217)^a^ | 360 |  | 131 (100–180)^a^ | 708 |  | 166 (115–330)^a^ | 472 |  | – | – |
| Sample 2 | 142 (102–195)^b^ | 85 |  | 142 (102–184)^a^ | 196 |  | 161 (116–275)^a^ | 133 |  | – | – |
| Adjusted estimated habitual iodine intake (μg/day)^4,5^ | 114 (102, 126) | 330 |  | 108 (100, 115)^6^ | 668 |  | 269 (229, 307)^7^ | 457 |  | – | – |
| No supplement users | 114 (102, 126) | 330 |  | – | – |  | 204 (178, 251) | 232 |  | – | – |
| Supplement users^8^ | – | 0 |  | – | – |  | 355 (316, 392) | 195 |  | – | – |
| Prevalence of inadequate iodine intake (%)^9^ | 5.4 (0.0, 14.6) | 330 |  | 1.9 (0.0, 7.7) | 668 |  | 12.0 (3.2, 24.7) | 457 |  | – | – |
| No supplement users | 5.4 (0.0, 14.6) | 330 |  | – | – |  | 31.0 (10.5, 41.1) | 232 |  | – | – |
| Supplement users | – | 0 |  | – | – |  | 0.2 (0.0, 4.0) | 198 |  | – | – |
| Prevalence of excessive iodine intake (%)^8^ | 0.0 (0.0, 0.2) | 330 |  | 0.0 (0.0, 0.0) | 668 |  | 5.0 (0.0, 9.4) | 457 |  | – | – |
| No supplement users | 0.0 (0.0, 0.2) | 330 |  | – | – |  | 2.0 (0.0, 4.5) | 232 |  | – | – |
| Supplement users | – | 0 |  | – | – |  | 3.4 (0.0, 10.7) | 198 |  | – | – |
| UNaC (mg/L)^1,2^ |  |  |  |  |  |  |  |  |  |  |  |
| Sample 1 | 3235 (2243–4476)^a^ | 359 |  | 3212 (2305–4038)^a^ | 715 |  | – | – |  | 1872 (927–3008) | 352 |
| Sample 2 | 3353 (2101–4411)^a^ | 96 |  | 2931 (2085–4087)^a^ | 209 |  | – | – |  | 1693 (868–2844) | 104 |
| Adjusted UNaC^4,5^ | 3342 (3184, 3513) | 335 |  | 3157 (3070, 3237) | 675 |  | – | – |  | 2011 (1872, 2143) | 343 |
| UNaC/UCC ratio (mg/g)^,2^ |  |  |  |  |  |  |  |  |  |  |  |
| Sample 1 | 4049 (2463–5693)^11,a^ | 347 |  | 3127 (2099–4395)^a^ | 703 |  | – | – |  | – | – |
| Sample 2 | 3288 (2026–5538)^b^ | 86 |  | 2944 (1942–4035)^a^ | 194 |  | – | – |  | – | – |
| Adjusted estimated habitual sodium excretion (mg/day)^4,5^ | 2350 (2205, 2506) | 319 |  | 2394 (2059–2770)^6^ | 673 |  | – | – |  | – | – |
| Tg (μg/L) | – | – |  | – | – |  | 26.0 (17.5–37.2) | 465 |  | 23.8 (15.5–35.3) | 347 |
| Prevalence of elevated Tg (%)^12^ | – | – |  | – | – |  | 17.0 | 79 |  | 13.3 | 46 |
| TSH (mU/L)^13^ | – | – |  | – | – |  | 1.8 (1.6–2.0) | 383 |  | 0.8 (0.6–1.1) | 352 |
| TT4 (nmol/L)^14^ | – | – |  | – | – |  | 71.5 (59.5–81.7) | 383 |  | 132.5 (33.7)^15^ | 351 |
| Prevalence of thyroid dysfunction (% [n])^16^ | – | – |  | – | – |  | 0.2 [1] | 383 |  | 6.3 [22] | 351 |

UIC, spot urinary iodine concentration; UCC*,* spot urinary creatinine concentration; UNaC*,* spot urinary sodium concentration; Tg*,* thyroglobulin.

^1^Values are median (interquartile range), all such values;

^2^Wilcoxson signed rank test was used to test differences between sample 1 and sample 2. Values with different superscript letters differed (*P*<0.05);

^3^*n*=40 outliers removed due to contamination with iodine;

^4^Values are median (bootstrap 95% CI), all such values;

^5^Adjusted distribution accounting for intra-individual variability using the SPADE method and the package “SPADE-RIVM” [2] in R [3];

^6^Adjusted distribution accounting for intra-individual variability using the Iowa State University Software for Intake Distribution Estimation [4] is 89 μg/day (IQR: 75–106, *n*=220) for children 6-8 years and 110 μg/day (IQR: 90–136, *n*=457) for children 9-12 years, prevalence of inadequate iodine intake is 10% and prevalence of excessive iodine intake is 0% (*n*=677) [1];

^7^*n*=5 outliers removed (iodine intakes >4’000 μg/day);

^8^Supplement user defined as taking dietary supplements containing 150 to 220 μg iodine/day;

^9^Percentage (bootstrapped 95% CI) of individuals with habitual intakes <AR of 65 μg/day for school-age children and 160 μg/day for pregnant women [5];

^10^Percentage of individuals with habitual intakes >UL of 450 μg/day for school-age children and 600 μg/day for pregnant women and from ESFA [6]. Applying the UL of 1’100 μg/day for pregnant women from NAM, the prevalence of excessive iodine intake was 0% in supplement users and non-users, respectively [5];

^11^*n*=7 outliers removed (sodium excretion >15’000 mg/day);

^12^Defined as Tg >43.5 μg/L [7];

^13^Using the machine and related Neonatal TSH/T4 kit NS2400 (Labsystems Diagnostics Oy, Vantaa, Finland) in 2020-2022 and GSP 2021-0010 (PerkinElmer, Turku, Finland) in 2015. Assay-specific normal reference range for non-pregnant adults are 0.1–3.7 nmol/L for NS2400 and GSP;

^14^Using the machine and related Neonatal TSH/T4 kit NS2400 (Labsystems Diagnostics Oy, Vantaa, Finland) in 2020-2022 and GSP 2021-0010 (PerkinElmer, Turku, Finland) in 2015. Assay-specific normal reference range for non-pregnant adults are 20–130 nmol/L for NS2400 and 65–165 nmol/L for GSP;

^15^Values are geomean (SD);

^16^Defined as abnormal TSH and/or TT4 (including subclinical and overt hypothyroidism, subclinical and overt hyperthyroidism, and isolated hypothyroxinaemia).

**Supplementary Table S3** Determinants of urinary iodine and creatinine concentrations in school-age children (*n=*362) in the 2020-2022 Swiss national study

|  | **UIC (μg/L)^1^** | *n* | *P*-value^2^ | *P*-value^3^ |  | **UCC (g/L)** | *n* | *P*-value^2^ | *P*-value^4^ |
| --- | --- | --- | --- | --- | --- | --- | --- | --- | --- |
| Median | 127 (87–194) | 362 |  |  |  | 0.9 (0.6–1.2) | 360 |  |  |
| Sex |  |  | 0.618 | – |  |  |  | 0.034 | 0.010 |
| Male | 127 (89–200) | 166 |  |  |  | 0.9 (0.6–1.3) | 165 |  |  |
| Female | 129 (80–194) | 172 |  |  |  | 0.8 (0.6–1.1) | 171 |  |  |
| Region |  |  | 0.030 | 0.003 |  |  |  | <0.001 | 0.151 |
| 1=Western (Romandie) | 145 (96–209) | 80 |  |  |  | 1.0 (0.8–1.3) | 79 |  |  |
| 2=Central-eastern | 137 (90–205) | 90 |  |  |  | 0.7 (0.1–1.1) | 90 |  |  |
| 3=Northwest and midland | 154 (108–234) | 16 |  |  |  | 0.8 (0.7–1.1) | 16 |  |  |
| 4=North-eastern | 104 (76–187) | 113 |  |  |  | 0.8 (0.5–1.1) | 113 |  |  |
| 5=Southern (Ticino) | 119 (84–171) | 63 |  |  |  | 0.9 (0.6–1.3) | 62 |  |  |
| Iodised salt |  |  | 0.325 | – |  |  |  | 0.167 | – |
| No | 101 (80–170) | 41 |  |  |  | – | – |  |  |
| Yes | 129 (89–196) | 266 |  |  |  | – | – |  |  |
| Daily dairy product consumption |  |  | 0.013 | – |  |  |  | 0.317 | – |
| No | 111 (79–163) | 105 |  |  |  | – | – |  |  |
| Yes | 136 (91–200) | 194 |  |  |  | – | – |  |  |
| Daily milk consumption |  |  | 0.009 | <0.001 |  |  |  | 0.788 | – |
| No | 114 (79–169) | 105 |  |  |  | – | – |  |  |
| Yes | 137 (95–204) | 194 |  |  |  | – | – |  |  |

UIC, spot urinary iodine concentration; UCC*,* spot urinary creatinine concentration.

^1^Values are median (interquartile range), all such values;

^2^*P*-values from a Mann-Whitney-U test (for two-categorical variables) or a Kruskal-Wallis followed by Mann-Whitney post hoc test with Bonferroni correction (for variables with more than two categories);

^3^*P*-values from multiple linear regression model with log UIC as the dependent variable and log UCC, log UNaC, region, daily milk consumption as independent variables (F(4,288)=38.560, *P*<0.001, R^2^=0.349);

^4^*P*-values from multiple linear regression model with log UCC as the dependent variable and age, sex, region as independent variables (F(3,331)=8.733, *P*<0.001, R^2^=0.07).

**Supplementary Table S4** Determinants of urinary iodine concentration, urinary creatinine concentration and thyroglobulin in pregnant women (*n=*513) in the 2020-2022 Swiss national study

|  | **UIC (μg/L)**^1^ | *n* | *P*-value^2^ | *P*-value^3^ | *P*-value iodine intakes^4^ | **UCC (g/L)** | *n* | *P*-value^2^ | *P*-value^5^ | **Tg (μg/L)** | *n* | *P*-value^2^ | *P*-value^6^ |
| --- | --- | --- | --- | --- | --- | --- | --- | --- | --- | --- | --- | --- | --- |
| Median | 97 (45–187) | 473 |  |  |  | 0.55 (0.3–1.0) | 472 |  |  | 26 (18–37) | 465 |  |  |
| Iodine-containing supplements |  |  | <0.001 | <0.001 | <0.001 |  |  | 0.113 | – |  |  | <0.001 | <0.001 |
| No | 81 (35–144) | 236 |  |  |  | 0.56 (0.3–0.9) | 236 |  |  | 29 (19–42) | 228 |  |  |
| Yes | 129 (68–218) | 197 |  |  |  | 0.53 (0.3–1.0) | 197 |  |  | 23 (16–32) | 195 |  |  |
| Trimester |  |  | 0.429 | – | – |  |  | 0.063 | – |  |  | 0.897 | – |
| 1 | 120 (48–235) | 56 |  |  |  | 0.80 (0.3–1.1) | 56 |  |  | 29 (20–37) | 51 |  |  |
| 2 | 97 (43–196) | 217 |  |  |  | 0.56 (0.3–1.0) | 217 |  |  | 26 (18–36) | 219 |  |  |
| 3 | 94 (47–171) | 178 |  |  |  | 0.49 (0.3–0.9) | 178 |  |  | 26 (17–40) | 173 |  |  |
| Educational level |  |  | 0.002 | 0.064 | 0.137 |  |  | 0.002 | 0.008 |  |  | 0.448 | 0.463 |
| 1=Compulsory schooling | 141 (76–220) | 18 |  |  |  | 0.90 (0.7–1.1) | 18 |  |  |  |  |  |  |
| 2=Apprenticeship | 120 (53–216) | 184 |  |  |  | 0.65 (0.3–1.1) | 184 |  |  |  |  |  |  |
| 3=Apprenticeship with vocational baccalaureate | 86 (48–196) | 23 |  |  |  | 0.46 (0.3–0.7) | 23 |  |  |  |  |  |  |
| 4=University of Applied Sciences/Technical College | 86 (39–163) | 93 |  |  |  | 0.54 (0.3–0.9) | 93 |  |  |  |  |  |  |
| 5=University | 77 (37–125) | 101 |  |  |  | 0.42 (0.2–0.8) | 101 |  |  |  |  |  |  |
| 6=Other | 100 (46–140) | 25 |  |  |  | 0.49 (0.3–1.0) | 25 |  |  |  |  |  |  |
| Nationality |  |  | 0.007 | 0.230 | 0.820 |  |  | <0.001 | 0.003 |  |  | 0.449 | 0.793 |
| Swiss | 94 (44–181) | 324 |  |  |  | 0.49 (0.2–0.9) | 324 |  |  | 26 (17–37) | 319 |  |  |
| Non-Swiss | 129 (66–231) | 104 |  |  |  | 0.75 (0.4–1.0) | 104 |  |  | 27 (19–38) | 104 |  |  |
| Region |  |  | <0.001 | 0.003 | 0.046 |  |  | <0.001 | 0.296 |  |  | <0.001 | 0.069 |
| 1=Western (Romandie) | 93 (45–141) | 25 |  |  |  | 0.81 (0.4–1.0) | 25 |  |  | 49 (34–62) | 25 |  |  |
| 2=Central-eastern | 102 (48–206) | 112 |  |  |  | 0.46 (0.3–0.8) | 112 |  |  | 28 (17–40) | 106 |  |  |
| 3=Northwest and midland | 172 (87–286) | 105 |  |  |  | 0.76 (0.4–1.2) | 104 |  |  | 23 (16–32) | 100 |  |  |
| 4=North-eastern | 80 (38–148) | 206 |  |  |  | 0.47 (0.2–0.9) | 206 |  |  | 25 (17–33) | 182 |  |  |
| 5=Southern (Ticino) | 59 (24–121) | 25 |  |  |  | 0.51 (0.3–0.9) | 25 |  |  | 35 (26–81) | 23 |  |  |
| Iodised salt |  |  | 0.054 | – | – |  |  | 0.148 | – |  |  | 0.222 | – |
| No | 113 (60–204) | 53 |  |  |  | 0.65 (0.3–0.9) | 53 |  |  | 26 (20–39) | 53 |  |  |
| Yes | 89 (41–177) | 333 |  |  |  | 0.47 (0.2–0.9) | 333 |  |  | 26 (16–36) | 326 |  |  |

UIC, spot urinary iodine concentration; UCC*,* spot urinary creatinine concentration; UNaC*,* spot urinary sodium concentration; Tg*,* thyroglobulin.

^1^Values are median (interquartile range), all such values;

^2^*P*-values from a Mann-Whitney-U test (for two-categorical variables) or a Kruskal-Wallis followed by Mann-Whitney post hoc test with Bonferroni correction (for variables with more than two categories);

^3^*P*-values from multiple linear regression models with log UIC as the dependent variable and log UCC, iodine-containing supplement use, education, nationality, region as independent variables (F(5,400)=79.454, *P*<0.001, R^2^=0.498);

^4^*P*-values from multiple linear regression models with log iodine intake as the dependent variable and log Tg, iodine-containing supplement use, education, nationality, region as independent variables (F(5,365)=11.897, P<0.001, R^2^=0.140);

^5^*P*-values from multiple linear regression models with log UCC as the dependent variable and age, pre-pregnancy BMI, education, nationality, region as independent variables (F(5,404)=6.659, *P*<0.001, R^2^=0.076);

^6^*P*-values from multiple linear regression models with log Tg as the dependent variable and age, iodine-containing supplement use, education, nationality and region as independent variables (F(5,368)=4.975, *P*<0.001, R^2^=0.063).

**References**

1. Andersson M, Hunziker S, Fingerhut R, Zimmermann M, Herter-Aeberli I (2019) Effectiveness of increased salt iodine concentration on iodine status: trend analysis of cross-sectional national studies in Switzerland. Eur J Clin Nutr 59 (2):581-593. doi:10.1007/s00394-019-01927-4

2. Dekkers A, Verkaik-Kloosterman J, van Rossum C, Ocké M (2014) SPADE, a new statistical program to estimate habitual dietary intake from multiple food sources and dietary supplements. J Nutr 144 (12):2083-2091. doi:10.3945/jn.114.191288

3. R Development Core Team (2021) R: A language and environment for statistical computing. 4.1.2. edn. R Foundation for Statistical Computing, Vienna, Austria

4. Dodd K (1996) A technical guide to C-SIDE. Dietary assessment research series report 9. CARD technical report 96-TR32. Center for Agricultural and Rural Development (CARD) at Iowa State University, Ames, Iowa

5. IOM (2001) Dietary reference intakes for vitamin A, vitamin K, arsenic, boron, chromium, copper, iodine, iron, manganese, molybdenum, nickel, silicon, vanadium, and zinc, vol Iodine. vol 8. National Academies Press (US), Washington (DC)

6. EFSA (2018) Summary of tolerable upper intake levels - version 4 (September 2018). European Food Safety Authority: Scientific Committee on Food and Scientific Panel on Dietetic Products, Nutrition and Allergies,

7. Stinca S, Andersson M, Weibel S, Herter-Aeberli I, Fingerhut R, Gowachirapant S, Hess S, Jaiswal N, Jukic T, Kusic Z, Mabapa N, Nepal A, San Luis T, Zhen J, Zimmermann M (2017) Dried blood spot thyroglobulin as a biomarker of iodine status in pregnant women. J Clin Endocrinol Metab 102 (1):23-32. doi:10.1210/jc.2016-2829
